# Exploration of the Pathogenesis and Treatment of Heart Failure

**DOI:** 10.31083/RCM49678

**Published:** 2026-06-29

**Authors:** Jordan M Tumes, Adit Ravishankar, Ismael H Khan, Ahmed R Gonnah, David H Roberts

**Affiliations:** ^1^University of Liverpool, L69 7ZX Liverpool, UK; ^2^Department of Internal Medicine, Pennsylvania Hospital, University of Pennsylvania Health System, Philadelphia, PA 19107, USA

**Keywords:** heart failure, systolic heart failure, diastolic heart failure, ventricular remodelling, inflammation, sodium-glucose cotransporter 2 inhibitors, precision medicine, drug therapy

## Abstract

Heart failure remains a major cause of morbidity and mortality worldwide despite substantial advances in pharmacological and device-based therapies. Distinct phenotypes, including heart failure with reduced ejection fraction, mildly reduced ejection fraction, and preserved ejection fraction, differ markedly in underlying pathophysiology, clinical presentation, and therapeutic responsiveness. Therefore, a deeper understanding of disease mechanisms is essential for guiding evolving management strategies and identifying novel therapeutic targets. This narrative review synthesizes contemporary evidence on the pathogenesis and management of heart failure, integrating mechanistic biology with phenotype-specific treatment effects and acute–chronic disease transitions to provide a translational framework for care. Key clinical trials, guideline documents, and mechanistic studies were identified through structured searches of major medical databases and review of international guideline updates, with emphasis on recent advances in disease-modifying therapies, inflammatory mechanisms, and emerging treatment strategies across heart failure phenotypes. Heart failure with reduced ejection fraction is characterized predominantly by myocyte loss, adverse ventricular remodeling, and neurohormonal activation, with robust evidence supporting combined disease-modifying pharmacotherapy and selected device-based interventions. In contrast, heart failure with preserved ejection fraction is driven largely by systemic comorbidities, endothelial dysfunction, and myocardial fibrosis, contributing to impaired ventricular compliance and limited evidence-based treatment options. Sodium–glucose cotransporter 2 inhibitors have demonstrated consistent reductions in symptoms and hospitalizations across the spectrum of ejection fractions, representing a major advance in management. However, translation of mechanistic insights into effective therapies has been variable. Broad anti-inflammatory strategies targeting cytokine pathways have yielded mixed or neutral outcomes. In contrast, more targeted approaches, including interleukin-1 inhibition, inflammasome modulation, and mitochondrial-directed therapies, show emerging but heterogeneous signals that warrant phenotype-specific evaluation. Heart failure is a heterogeneous syndrome that requires biologically informed, phenotype-specific approaches. Thus, by linking dominant mechanisms to therapeutic response and highlighting the limitations of current evidence, this review aims to advance understanding beyond descriptive summaries and to outline priorities for future precision-oriented heart failure care.

## 1. Introduction

Heart failure (HF) is a recognised major public health issue worldwide. Recent European Society of Cardiology (ESC) Atlas and Global Burden of Disease reports find approximately 64 million people affected globally [1]. An aging population and modifiable co-morbidities contribute to the rising incidence of this multifactorial disease [2].

HF is a leading driver of hospital admissions and readmissions in older adults. A recent analysis estimated 1.6% of the UK population living with HF with 190,798 new cases annually [3]. Similar national British Heart Foundation report prevalence in that 1–2% range [4]. Globally these figures range from 1–14% and are expected to rise by 46% by 2030 [5]. Age-adjusted HF mortality has not fallen as fast as other cardiovascular disease (CVD) categories in some analyses; mortality and hospital burden are concentrated in older and multimorbid patients [4]. Global chronic heart failure cohorts demonstrate one-year mortality rates of 15–25% and five-year mortality rates below 50% despite therapeutic advancements [5].

Acute heart failure is a common cause of admission to hospital (over 67,000 admissions in England and Wales per year) and is the leading cause of hospital admission in people 65 years or older in the UK [6]. Large National Health Service (NHS) data analyses (2019–2022 admissions cohorts) show substantial in-hospital and early post-discharge morbidity and high readmission rates. Following a 33% rise in HF admissions between 2013/14 and 2018/19 (British Heart Foundation, 2019), from 2019 to 2021, there was a reduction in HF admissions citing the swift implementation of newer guideline-recommended medical therapies [7]. Beyond its impact on survival, heart failure imposes a substantial burden of debilitating symptoms including dyspnoea, fatigue, and exercise intolerance that markedly impair health-related quality of life and functional independence, outcomes that increasingly define therapeutic success from the patient’s perspective.

A universal HF definition developed and endorsed by Heart failure societies of America, Canada, Europe, Japan, India, China, Australia and New Zealand defines HF as a clinical syndrome with current or prior symptoms and/or signs (e.g., elevated jugular venous pressure, pulmonary crackles, peripheral oedema) caused by a structural and/or functional cardiac abnormality and corroborated by elevated natriuretic peptide levels or objective evidence of cardiogenic pulmonary or systemic congestion by diagnostic modalities [8].

Heart failure is increasingly recognised not as an isolated cardiac disorder, but as a systemic, multisystem syndrome driven by complex interactions between the heart and peripheral organs. A growing body of evidence highlights the central role of extracardiac dysfunction including renal impairment, skeletal muscle abnormalities, metabolic liver disease, pulmonary vascular remodelling, and adipose tissue dysregulation in contributing to myocardial stress, impaired cardiac reserve, and symptom burden. These processes are closely linked to common comorbidities such as obesity, diabetes, and ageing, which promote systemic inflammation, endothelial dysfunction, and metabolic derangements that converge on the myocardium. This paradigm represents a shift away from a purely cardio-centric model toward an integrated cardiometabolic framework of pathophysiology.

The objective of this narrative review is to synthesise and integrate contemporary evidence on the pathogenesis and management of heart failure across and within phenotypic subgroups, with a particular focus on the mechanistic pathways that underpin disease progression and therapeutic response on traditional and contemporary endpoints. By integrating these insights with recent advances in guideline-directed and emerging therapies, this review aims to clarify why treatment efficacy differs across heart failure phenotypes and to highlight areas where targeted, mechanism and symptom-based interventions may address persistent unmet clinical need.

## 2. Classification of Heart Failure

### 2.1 Ejection Fraction-Based

Left ventricular ejection fraction (LVEF) remains the cornerstone for classifying heart failure, as it correlates strongly with both underlying pathophysiology and response to therapy. Echocardiography is the standard method for measuring LVEF, with cardiac MRI reserved for cases requiring more precise volumetric assessment or tissue characterisation. Based on current European and UK National Institute for Health and Care Excellence (NICE) guidelines, heart failure is categorised as: heart failure with reduced ejection fraction (HFrEF; LVEF ≤40%), heart failure with mildly or moderately reduced ejection fraction (HFmrEF; LVEF 41–49%), heart failure with preserved ejection fraction (HFpEF; LVEF ≥50%), and heart failure with improved ejection fraction (HFimpEF), the latter describing patients with prior HFrEF whose ejection fraction (EF) has improved to >40% with optimal therapy (Table 1).

**Table 1.** **HF classification types, categories, key features and clinical focus**.

Classification type	Categories	Key features	Clinical focus
Ejection fraction (EF)	HFrEF (≤40%), HFmrEF (41–49%), HFpEF (≥50%), HFimpEF (improved)	Based on left ventricular systolic function. Defines HF subtype and guide’s therapy.	Links EF to pathophysiology, treatment, and prognosis; cornerstone of ESC/NICE classification.
Time course	Acute vs Chronic	Acute = rapid onset/worsening.	Distinguishes acute decompensation vs long-term management.
		Chronic = long-standing stable or recurrent.	
Anatomical side	Left vs Right	Left = pulmonary congestion.	Identifies dominant ventricle and congestion pattern.
		Right = systemic congestion, oedema, ascites.	
Hemodynamic load	Pressure vs Volume Overload	Pressure overload (HTN) → concentric LVH. Volume overload (regurgitation, anaemia) → dilation.	Links pathophysiology to remodelling and EF pattern.
Functional capacity (NYHA)	Classes I–IV	I = no limitation → IV = symptoms at rest.	Grades severity and prognosis, guides therapy selection.
Disease stage (ACC/AHA)	Stages A–D	A = risk only; B = structural disease; C = symptomatic; D = refractory advanced HF.	Highlights prevention, early intervention, and advanced care needs.

HF, heart failure; HFrEF, heart failure with reduced ejection fraction; HFmrEF, heart failure with mildly or moderately reduced ejection fraction; HFpEF, heart failure with preserved ejection fraction; HFimpEF, heart failure with improved ejection fraction; ESC, European Society of Cardiology; NICE, National Institute for Health and Care Excellence; HTN, hypertension; LVH, left ventricular hypertrophy; NYHA, New York Heart Association; ACC, American College of Cardiology; AHA, American Heart Association.

The pathophysiological mechanisms differ significantly across these EF-based categories. HFrEF is primarily a disorder of systolic dysfunction, resulting from loss of viable cardiomyocytes due to ischaemic injury, myocarditis, or dilated cardiomyopathy. This leads to eccentric remodelling, chamber dilation, and wall thinning, and progressive contractile failure (Narayan et al., 2023 [9]). By contrast, HFpEF is characterised by diastolic dysfunction, impaired ventricular relaxation, increased myocardial stiffness, and concentric hypertrophy, typically driven by systemic comorbidities such as hypertension (HTN), obesity, and diabetes, which are associated with a state of chronic systemic inflammation. This inflammatory state, marked by elevated circulating pro-inflammatory cytokines (including interleukin-6 [IL-6] and tumour necrosis factor-α [TNF-α]), oxidative stress, and endothelial activation promotes coronary microvascular inflammation and endothelial dysfunction, ultimately leading to myocardial fibrosis and reduced ventricular compliance rather than primary myocyte loss [10].

HFmrEF represents an intermediate phenotype, sharing features of both HFrEF and HFpEF, and often responds to therapies proven effective in HFrEF. HFimpEF, on the other hand, reflects reverse remodelling and functional recovery after guideline-directed therapy, although patients remain at risk of relapse if treatment is withdrawn [11].

The clinical importance of EF-based classification lies in its prognostic and therapeutic implications. Only HFrEF currently has multiple disease-modifying therapies (angiotensin receptor-neprilysin inhibitors (ARNIs), angiotensin converting enzyme (ACE) inhibitors, angiotensin receptor blockers (ARBs), beta-blockers, mineralocorticoid receptor antagonists (MRAs), sodium-glucose cotransporter 2 inhibitors (SGLT2 inhibitors)) with robust evidence for mortality reduction. In contrast, HFpEF management has traditionally focused on symptom control and optimisation of comorbidities. However, recent large-scale trials have demonstrated that SGLT2 inhibitors confer meaningful outcome benefits in this population. Specifically, the EMPEROR-Preserved and DELIVER trials showed a significant reduction in the composite risk of worsening heart failure or cardiovascular death among patients with HFpEF (Table 1) [12].

### 2.2 Acute vs Chronic HF

Heart failure can be classified by time course and acuity into acute and chronic presentations, which differ markedly in pathophysiology and management. Acute heart failure (AHF) refers to a rapid onset or worsening of symptoms and signs of HF, often leading to hospital admission. It may represent new-onset HF (de novo), such as after myocardial infarction (MI), or acute decompensation of previously chronic HF (ADHF). AHF is commonly triggered by infection, arrhythmia, ischaemia, poor medication adherence, or renal dysfunction. It carries high short-term mortality (up to 10% during hospital admission) and is a major driver of hospital resource use in the UK and Europe [13].

Chronic heart failure (CHF), in contrast, is a long-standing condition with periods of relative stability interspersed with episodes of decompensation. It reflects sustained neurohormonal activation and structural remodelling. Management focuses on optimising guideline-directed medical therapy (GDMT), comorbidity control, and patient self-care. Chronic HF prevalence continues to rise, reflecting improved survival after acute events and population ageing (Table 1) [14].

### 2.3 Left vs Right HF

Left-sided HF results from failure of the left ventricle to adequately pump or fill, leading to pulmonary congestion (pulmonary venous hypertension) and breathlessness. The most common causes are ischaemic heart disease, hypertension, and valvular disorders such as aortic stenosis or mitral regurgitation. Pulmonary venous hypertension can lead to secondary pulmonary arterial hypertension, with consequent right ventricular hypertrophy and dilatation [15].

Right-sided HF arises when the right ventricle fails to maintain adequate pulmonary circulation, leading to systemic venous congestion, peripheral oedema, hepatomegaly, and ascites. It may develop secondary to left-sided HF, chronic lung disease (Chronic Obstructive Pulmonary Disease (COPD), pulmonary hypertension), or right-sided structural disease (tricuspid or pulmonary valve pathology). Cor pulmonale, defined as an alteration in the structure and function of the right ventricle is caused by a primary disorder of the respiratory system associated with pulmonary arterial hypertension. Recent evidence highlights that right ventricular dysfunction is an independent prognostic marker in both HFrEF and HFpEF, with emerging imaging (e.g., right ventricular strain on echocardiography or Cardiac Magnetic Resonance) refining risk assessment (Table 1) [16].

### 2.4 Haemodynamic Overload (Pressure vs Volume)

HF can arise from two main types of haemodynamic stress, pressure overload and volume overload, each producing distinct patterns of ventricular remodelling. Pressure overload, seen in long-standing hypertension or aortic stenosis, leads to concentric left ventricular hypertrophy (LVH). The myocardium thickens to overcome the increased afterload, resulting in preserved or initially increased EF but impaired diastolic filling.

Volume overload, such as in valvular regurgitation or chronic anaemia, causes ventricular dilation and eccentric hypertrophy as the ventricle adapts to increased preload [17,18]. Over time, this can lead to systolic dysfunction and reduced EF. These adaptations link the type of haemodynamic load directly to the pattern of ventricular remodelling and the eventual heart failure phenotype (HFpEF vs HFrEF) (Table 1).

### 2.5 NYHA Functional Classes & American College of Cardiology (ACC)/American Heart Association (AHA) Stages

The New York Heart Association (NYHA) system is entirely symptom-based, grading patients according to how their daily activities are limited by breathlessness, fatigue, or palpitations. This approach captures the functional impact of heart failure on quality of life and allows clinicians to monitor changes over time or in response to therapy. Importantly, NYHA class correlates with health-related quality-of-life measures such as the Kansas City Cardiomyopathy Questionnaire (KCCQ) and independently predicts hospitalisation and mortality, underscoring the close relationship between symptom burden, patient experience, and prognosis. Although subjective and associated with inter-observer variability, the NYHA classification remains clinically valuable because it reflects the lived experience of disease and guides therapeutic decision-making and trial eligibility.

In contrast, the ACC/AHA stages describe the progressive trajectory of disease from individuals at risk (Stage A) to those with advanced, refractory heart failure (Stage D) emphasising structural cardiac changes and the potential for prevention (Table 1). While primarily focused on pathophysiology and disease progression, the staging framework also highlights opportunities for early intervention to prevent the development or worsening of symptomatic limitations that ultimately impair functional capacity and quality of life. Together, these complementary classification systems illustrate that heart failure assessment encompasses not only structural and biological progression but also the dynamic evolution of symptoms and functional status that matter most to patients [19].

## 3. Pathogenesis of Heart Failure

### 3.1 Neurohormonal Activation

Neurohormonal activation refers to the sustained stimulation of multiple circulating and tissue-level hormonal systems in response to reduced cardiac output or arterial under-filling. These systems are principally the sympathetic nervous system (SNS), the renin-angiotensin-aldosterone system (RAAS), vasopressin (ADH), and counter-regulatory natriuretic peptides (BNP, NT-proBNP). While initially compensatory (raise blood pressure to preserve perfusion), they can lead to chronic activation and become maladaptive, driving tissue remodelling, fibrosis, arrhythmia and organ dysfunction. This mechanistic concept is central to modern HF pathophysiology and therapy [20].

In HFrEF, sustained activation of the SNS and RAAS constitutes the principal maladaptive driver of disease progression. Persistent neurohormonal stimulation promotes cardiomyocyte apoptosis, adverse ventricular remodelling, myocardial fibrosis, and arrhythmogenesis, thereby directly contributing to systolic dysfunction and mortality [21]. The consistent survival benefit observed with β-blockers, ACE inhibitors, ARBs, MRAs, and ARNIs strongly supports the centrality of neurohormonal dysregulation in this phenotype [21].

In contrast, although neurohormonal activation is detectable in HFpEF, it appears less dominant in driving disease progression. Large, randomised trials of RAAS inhibition and β-blockade in HFpEF have generally demonstrated neutral or modest effects on mortality, suggesting that neurohormonal activation in this setting may represent a secondary adaptive response rather than a primary therapeutic target [10]. This divergence highlights fundamental biological differences between heart failure phenotypes and underscores the importance of mechanism-aligned therapy.

#### 3.1.1 Sympathetic Nervous System

Baroreceptor unloading and reduced cardiac output increase central sympathetic outflow and catecholamine (norepinephrine) release. The persistent β-adrenergic stimulation produces tachycardia, increased myocardial oxygen demand, adrenergic-mediated myocyte apoptosis, receptor down-regulation/desensitisation, and arrhythmogenic electrical instability. Elevated plasma norepinephrine predicts worse prognosis [21].

#### 3.1.2 Renin-Angiotensin-Aldosterone System

Reduced renal perfusion and sodium delivery trigger renin release leading to angiotensin II formation and additional aldosterone secretion. Angiotensin II causes vasoconstriction, hypertrophy by up-regulating pro-growth and pro-fibrotic gene expression**, **inflammation through oxidative stress, cytokine release (IL-6, TNF-α), leukocyte recruitment, and endothelial activation stimulating fibrosis. Aldosterone promotes sodium/water retention and myocardial/interstitial fibrosis. Chronic RAAS activation worsens remodelling and heart failure progression (Table 2) [22].

**Table 2.** **Consequences of Angiotensin II-induced inflammation, target tissue and pathological consequences**.

Target tissue	Inflammatory effect	Pathological consequence
Myocardium	Cytokine release (IL-6, TNF-α), fibroblast activation, leukocyte infiltration	Fibrosis, hypertrophy, contractile dysfunction
Vascular wall	Endothelial activation, ROS, macrophage infiltration	Atherosclerosis, vascular stiffness
Kidney	Mesangial inflammation, fibrosis	Renal dysfunction, further RAAS activation

IL-6, interleukin-6; TNF-α, tumour necrosis factor-α; ROS, reactive oxygen species; RAAS, renin-angiotensin-aldosterone system.

#### 3.1.3 Anti-Diuretic Hormone

Angiotensin II directly stimulates ADH neurons in the hypothalamus, creating a positive feedback loop between RAAS and ADH systems. ADH increases water reabsorption and vasoconstriction. Chronic elevation contributes to hyponatraemia, volume overload and increased vascular resistance, markers of poor prognosis in HF. In heart failure, ADH is persistently elevated due to non-osmotic baroreceptor activation. Plasma ADH levels are markedly elevated in chronic and decompensated HF and correlate with disease severity and mortality. Hyponatraemia (<135 mmol/L) in HF is a strong independent predictor of poor outcomes, often reflecting excessive ADH activity [23].

#### 3.1.4 Natriuretic Peptide System

The natriuretic peptide (NP) system acts as an essential counter-regulatory mechanism to maladaptive neurohormonal activation in HF. The principal peptides are atrial natriuretic peptide (ANP), B-type natriuretic peptide (BNP), and C-type natriuretic peptide (CNP). ANP and BNP are secreted from cardiac myocytes in response to increased wall stress, while CNP is produced by vascular endothelium [24]. NPs promote natriuresis, diuresis, and vasodilation through activation of guanylyl cyclase-coupled receptors (NPR-A/B), increasing cyclic GMP signalling. This cascade reduces preload and afterload, inhibits renin, aldosterone, and vasopressin release, and exerts anti-hypertrophic and anti-fibrotic effects on the myocardium. The NP system thus directly opposes the RAAS, SNS, ADH and endothelin pathways.

In chronic HF, persistent ventricular stretch leads to elevated BNP and ANP secretion, making plasma BNP or NT-proBNP key biomarkers of cardiac wall stress and prognosis. However, despite high circulating levels, patients often exhibit natriuretic peptide resistance, caused by enhanced degradation by neprilysin, downregulation of NP receptors, and counteracting RAAS/SNS activation. Consequently, the beneficial vasodilatory and natriuretic actions are blunted, perpetuating fluid overload and remodelling. The NP system represents the heart’s intrinsic defence against volume and pressure overload. In HF, persistent activation initially compensates but ultimately becomes insufficient due to receptor desensitization and enzymatic degradation. Restoring NP effectiveness through neprilysin inhibition and cGMP modulation now forms a central component of evidence-based heart failure management, improving survival and quality of life across HF phenotypes.

### 3.2 Genetic and Molecular Mechanisms

Genetic predisposition determines myocardial resilience, repair, and remodelling. Genetic causes are most obvious in younger or familial cardiomyopathies but contribute to risk across the HF spectrum (including non-ischaemic and HFpEF phenotypes). Recent large-scale genomic studies have expanded the landscape from rare high-penetrance variants to common polygenic contributions [25]. Genome-wide association studies (GWAS) and large meta-analyses now identify dozens of loci associated with HF and its subtypes (HFrEF, HFpEF), implicating pathways in cardiac development, fibrosis, metabolism and immune regulation [26]. While genetic discoveries were historically viewed as mechanistic insights, their clinical relevance in heart failure is increasingly recognised. In dilated cardiomyopathy (DCM), common pathogenic variants involve nuclear envelope dysfunction and truncating *TTN* gene mutations. In Hypertrophic cardiomyopathy (HCM), sarcomeric dysfunction has been identified as a primary driver. They are associated with heightened arrhythmic risk, progressive conduction disease, and adverse prognosis independent of left ventricular ejection fraction [27].

GWAS can stratify patients at elevated lifetime risk and identify pathogenic pathways. These genotype-phenotype correlations have direct implications for risk stratification, including earlier consideration of implantable cardioverter-defibrillator (ICD) therapy in selected patients who may not meet conventional ejection fraction-based thresholds. Contemporary cardiomyopathy guidelines now recommend incorporating genetic findings into decisions regarding device therapy and family screening [28]. Beyond device implantation, genomic information refines prognostication and surveillance strategies. *LMNA* gene mutation carriers, for example, exhibit high lifetime arrhythmic risk warranting intensified rhythm monitoring, while cascade genetic screening enables identification of asymptomatic relatives who may benefit from early evaluation and follow-up [29].

#### 3.2.1 Sarcomeric Dysfunction

Mutations alter contractile force generation or kinetics, provoking compensatory hypertrophy and energetic stress. Sarcomeric gene mutations (*MYH7*, *MYBPC3*, *TNNT2*) underlie HCM and can progress to HF via hypertrophy, microvascular ischemia and fibrosis; desmosomal gene variants (*PKP2*, *DSP*) drive arrhythmogenic cardiomyopathies.

#### 3.2.2 Proteostasis and TTN Biology

*TTN* truncations weaken passive stiffness and sarcomere integrity, predisposing to dilation under stress. *TTN* truncating variants are the single most frequent genetic cause of DCM and substantially increase risk of systolic HF. *TTN* variants account for ~10–20% of idiopathic DCM in many cohorts [26].

#### 3.2.3 Nuclear Envelope Dysfunction (LMNA)

While not yet fully understood, the current mechanical hypothesis suggests that the destruction of the nuclear layer increases nuclear fragility and sensitivity to mechanical stress, making myocardial tissue more susceptible to pathological effects. *LMNA* mutations display high rates of sudden cardiac death due to malignant arrhythmias [29]. The *LMNA* gene is the second most frequently mutated gene in DCM [26].

### 3.3 Inflammation and Oxidative Stress

Inflammation and oxidative stress are central, interacting drivers of myocardial remodelling, dysfunction and clinical progression in HF. Cardiac injury (ischaemia, pressure/volume overload, genetic cardiomyopathies) activates innate immune signalling in cardiomyocytes, fibroblasts and endothelial cells [20]. Pro-inflammatory cytokines (IL-1β, IL-6, TNF-α) and chemokines (MCP-1), recruit leukocytes and activate fibroblasts in response to damage-associated molecular patterns (DAMPs) and toll-like receptors. Neurohormonal system activation stimulates mitochondrial reactive oxygen species (ROS) production which propagates oxidative stress and promotes myocyte apoptosis, impairs excitation-contraction (EC) coupling, and stimulates profibrotic pathways. The result is an anticipatory feed-forward cycle: inflammation and oxidative stress → fibrosis, hypertrophy and adverse remodelling → worse haemodynamics and further inflammation.

Clinically, systemic and myocardial inflammatory markers (high sensitivity C-reactive protein (CRP), IL-1, IL-6, TNF-α, myeloperoxidase (MPO)) and oxidative markers correlate with HF severity and prognosis. This mechanistic link of inflammatory and oxidative markers motivated trials of immune-modulating therapies. Inflammatory signalling plays divergent roles across heart failure phenotypes. In HFrEF, inflammation is typically activated downstream of myocardial injury and neurohormonal excess, acting as an amplifier of adverse remodelling rather than an initiating mechanism. This may explain why broad anti-cytokine strategies, including tumour necrosis factor inhibition, have failed to show definitive clinical outcome benefits within this phenotype despite strong associations between inflammatory biomarkers and prognosis. By contrast, HFpEF is increasingly conceptualised as a systemic, comorbidity-driven inflammatory syndrome. Obesity, diabetes, and hypertension promote chronic low-grade inflammation, endothelial dysfunction, coronary microvascular rarefaction, and profibrotic signalling, leading predominantly to myocardial stiffening and diastolic dysfunction [10]. This mechanistic framework provides a plausible explanation for the limited efficacy of traditional neurohormonal therapies in HFpEF and supports continued exploration of targeted metabolic and anti-inflammatory strategies.

### 3.4 Calcium Handling and Contractile Dysfunction

Calcium handling is central to normal cardiac excitation-contraction coupling and is tightly regulated by a network of intracellular proteins that control calcium release, reuptake, and storage within the sarcoplasmic reticulum (SR). In a healthy cardiomyocyte, electrical activation triggers ryanodine receptor (RyR2)-mediated calcium release from the SR, enabling contraction, while relaxation occurs when calcium is resequestered into the SR by the ATP-dependent pump SERCA2a [30].

These intrinsic mechanisms are profoundly disrupted in HFrEF, making calcium cycling one of the most important cellular abnormalities underpinning impaired contractility, arrhythmia, and myocardial remodelling and a key therapeutic target. A hallmark of HFrEF is reduced SERCA2a expression and activity, which slows diastolic calcium reuptake (impaired lusitropy) and diminishes SR calcium stores. Additionally, RyR2 channels become hyperphosphorylated and “leaky”, often through chronic β-adrenergic stimulation and oxidative stress. This diastolic calcium leak further depletes SR calcium stores, reduces systolic calcium release, and promotes arrhythmogenesis.

### 3.5 Integrated Multiorgan Model

Heart failure is increasingly recognised as a multisystem disorder involving complex interactions between the heart and peripheral organs, including the kidney, skeletal muscle, liver, lung, adipose tissue and gastrointestinal tract [10,31]. These extracardiac abnormalities contribute to myocardial stress via a range of mechanisms as detailed below, however, their role differs fundamentally between heart failure phenotypes. In HFpEF, cardiometabolic comorbidities, including obesity, ageing and diabetes act as primary drivers of disease, initiating a systemic pro-inflammatory state that promotes coronary microvascular dysfunction, myocardial fibrosis, and diastolic stiffness [31,32]. In contrast, HFrEF is typically initiated by primary myocardial injury due to ischemia or cardiomyopathy, with multiorgan dysfunction emerging as a secondary consequence of reduced cardiac output and chronic neurohormonal activation, further exacerbating disease progression [31,33]. Consequently, while multiorgan involvement is central to both syndromes, HFpEF is best characterised as a systemic cardiometabolic disorder, whereas HFrEF represents a cardiac-centric disease with secondary systemic involvement.

### 3.6 Cardiorenal Interactions

Renal dysfunction is highly prevalent in HFpEF and contributes through bidirectional cardiorenal mechanisms. Chronic kidney disease (CKD) promotes systemic inflammation, oxidative stress, and endothelial dysfunction, which accelerate myocardial remodelling and diastolic stiffness [10]. Neurohormonal activation drives sodium retention, plasma volume expansion, and elevated filling pressures, increasing myocardial wall stress [34]. Reduced nitric oxide bioavailability further impairs both renal and coronary microvascular function. In addition, uremic toxins such as indoxyl sulfate directly induce cardiomyocyte oxidative stress, mitochondrial dysfunction, and fibroblast activation, promoting myocardial fibrosis and stiffness [34]. These processes underpin the cardiorenal syndrome and amplify HFpEF progression.

### 3.7 Skeletal Muscle Dysfunction and Peripheral Myopathy

Exercise intolerance in HFpEF is strongly driven by peripheral limitations. Skeletal muscle exhibits reduced mitochondrial density, impaired oxidative phosphorylation, and diminished capillary density, limiting oxygen extraction during exertion [35]. Abnormal substrate utilisation including impaired fatty acid oxidation and branched-chain amino acid metabolism leads to metabolic inflexibility and early lactate accumulation [36]. Mitochondrial respiratory dysfunction correlates closely with reduced peak VO_2_, a key determinant of prognosis [35]. Systemic inflammation, insulin resistance, and adipokine imbalance further promote sarcopenia, reduced type I oxidative fibres, and impaired muscle perfusion, reinforcing exercise intolerance independent of cardiac output.

### 3.8 Liver Dysfunction and the Heart-Liver Axis

Metabolic dysfunction-associated steatotic liver disease (MASLD), formerly known as non-alcoholic fatty liver disease (NAFLD) is highly prevalent in HFpEF and contributes via shared metabolic and inflammatory pathways. The steatotic liver secretes hepatokines, cytokines, and extracellular vesicles that promote endothelial dysfunction, myocardial inflammation, and fibrotic signalling [37]. Hepatic insulin resistance increases circulating triglyceride-rich lipoproteins and free fatty acids, driving lipotoxicity and myocardial energetic impairment [38]. Advanced fibrosis alters hepatic vascular resistance and venous capacitance, impairing preload reserve and cardiovascular adaptability during exercise [38]. These mechanisms support a bidirectional heart–liver axis in HFpEF.

### 3.9 Pulmonary and Pulmonary Vascular Dysfunction

Elevated left ventricular filling pressures in HFpEF are transmitted retrogradely to the pulmonary circulation, leading to pulmonary venous hypertension, endothelial dysfunction, and capillary stress failure [39]. Chronic exposure to elevated pressures promotes pulmonary vascular remodelling, characterized by medial hypertrophy, intimal fibrosis, and reduced vascular compliance. Over time, this process may progress from isolated post-capillary pulmonary hypertension to combined pre- and post-capillary pulmonary hypertension (CpcPH), reflecting superimposed pulmonary arteriolar remodelling and increased pulmonary vascular resistance [40]. This transition represents a key pathophysiological step associated with worse prognosis.

The resulting increase in pulmonary vascular load imposes a significant burden on the right ventricle, leading to right ventricular–pulmonary arterial (RV–PA) uncoupling, reduced right ventricular contractile reserve, and ultimately right ventricular dysfunction. In advanced stages, this may resemble a form of secondary cor pulmonale, although in HFpEF this is driven primarily by left heart disease rather than intrinsic pulmonary pathology. Impaired pulmonary vascular reserve, reduced diffusion capacity, and ventilation–perfusion mismatch further limit oxygen uptake during exercise, contributing to exertional dyspnoea and reduced functional capacity. Additionally, right ventricular dysfunction exacerbates systemic venous congestion, creating a feedback loop that further worsens cardiac and multiorgan function [10].

### 3.10 Obesity and the Adipose Tissue–Heart Axis

Obesity is a central driver of HFpEF and represents a distinct clinical phenotype characterized by systemic inflammation, volume overload, and metabolic dysregulation [41]. Visceral adiposity, rather than overall BMI, appears particularly important, supporting the concept of a visceral adipose tissue-heart axis. Adipose tissue in obesity undergoes phenotypic transformation, shifting from a metabolically protective to a pro-inflammatory and endocrine-active organ. This is characterized by increased secretion of maladaptive adipokines (e.g., leptin, resistin) and reduced cardioprotective adiponectin, promoting endothelial dysfunction and myocardial fibrosis [42,43].

Adipokines are bioactive signalling molecules secreted predominantly by adipose tissue that exert endocrine and paracrine effects on the heart, vasculature, and kidneys. Leptin, which is markedly elevated in obese individuals, promotes myocardial hypertrophy, interstitial fibrosis, and endothelial dysfunction through activation of pro-inflammatory signalling cascades and stimulation of aldosterone secretion [44]. Resistin has similarly been associated with increased cardiovascular risk through its pro-inflammatory and pro-fibrotic properties, whilst circulating levels correlate with HF severity and adverse cardiac events [45]. Adiponectin, by contrast, normally confers anti-inflammatory, anti-hypertrophic, and insulin-sensitising effects on the myocardium; however, its expression is suppressed in the setting of visceral adiposity, removing an important cardioprotective influence [46].

A recent unifying framework has proposed that the expansion and biological transformation of visceral adipose tissue drive HFpEF pathogenesis through this adipokine imbalance, providing a mechanistic explanation for the strong epidemiological association between obesity and HFpEF that extends beyond haemodynamic loading alone [46]. Notably, central obesity or excess visceral adiposity is present in the vast majority of patients with established HFpEF, and the severity of haemodynamic abnormalities tracks with the degree of adiposity [46]. These observations suggest that adipokine-mediated signalling may represent both a modifiable risk pathway and a potential therapeutic target in the cardiometabolic HFpEF phenotype, although translation into clinical interventions remains at an early stage.

Obesity also drives neurohormonal and renal alterations, including aldosterone excess, natriuretic peptide deficiency, and renal sodium retention, resulting in plasma volume expansion and elevated filling pressures [42]. Similarly, cardiac lipotoxicity mediated by accumulation of triglycerides and free fatty acids induces mitochondrial dysfunction, oxidative stress, and cardiomyocyte apoptosis [42]. Epicardial and perivascular adipose depots exert direct paracrine effects on the myocardium and coronary microcirculation, promoting microvascular inflammation, reduced NO bioavailability, and extracellular matrix remodelling [43]. Importantly, excess adiposity is strongly associated with increased filling pressures, impaired cardiac reserve, and reduced exercise capacity, even independent of insulin resistance, highlighting adiposity as a primary driver of HFpEF pathophysiology [47].

### 3.11 Gut Microbiota Dysbiosis

Gut microbiota dysbiosis has emerged as a further contributor to HF pathogenesis through what has been termed the “gut hypothesis of heart failure” [48]. In chronic HF, reduced cardiac output and venous congestion lead to splanchnic hypoperfusion, intestinal mucosal ischaemia, and bowel wall oedema. Sandek et al. [49] and colleagues demonstrated that patients with chronic HF exhibit significantly increased bowel wall thickness, elevated intestinal permeability, and greater concentrations of adherent mucosal bacteria compared with healthy controls. This impaired intestinal barrier facilitates translocation of bacteria and bacterial products, notably lipopolysaccharide (LPS), into the systemic circulation, triggering inflammatory signalling [50]. Additionally, gut microbial metabolism of dietary choline and L-carnitine leads to elevated circulating of Trimethylamine N-oxide (TMAO), which has been independently associated with adverse prognosis in HF populations, with proposed mechanisms including activation of inflammasomes, promotion of myocardial fibrosis, impaired mitochondrial energetics, and endothelial dysfunction [51].

Compositional analyses of the gut microbiome in HF cohorts have consistently identified reductions in butyrate-producing commensals and increases in potentially pathogenic bacterial species, a pattern that may further compromise mucosal integrity and perpetuate systemic inflammation [52]. The relationship between the gut and the heart in HF is therefore bidirectional: cardiac dysfunction disrupts intestinal homeostasis, whilst gut-derived inflammatory mediators and metabolites exacerbate myocardial remodelling and disease progression. Although these mechanistic links are increasingly well characterised in preclinical and observational studies, interventional evidence from targeted microbiome-modulating strategies in HF populations remains limited, and further phenotype-specific clinical trials are warranted.

### 3.12 Ageing and Cellular Senescence

Ageing is the strongest non-modifiable risk factor for HFpEF, exerting direct effects on vascular, immune, and myocardial biology, whereas in HFrEF it acts predominantly as a susceptibility factor through its association with ischemic and structural heart disease. A central mechanism is cellular senescence, in which aged endothelial cells, immune cells, and fibroblasts adopt a senescence-associated secretory phenotype (SASP), characterized by chronic release of pro-inflammatory cytokines, growth factors, and proteases. This promotes systemic inflammation, endothelial dysfunction, and myocardial fibrosis [53].

Ageing is also associated with microvascular rarefaction and impaired endothelial nitric oxide signalling, leading to reduced myocardial perfusion reserve and increased cardiomyocyte stiffness. Increased oxidative stress further impairs NO–cGMP–PKG signalling, a key pathway regulating myocardial relaxation. At the cardiomyocyte level, ageing leads to mitochondrial dysfunction, impaired calcium handling, and reduced ATP availability, which directly impair diastolic relaxation and increase susceptibility to energetic stress. In addition, ageing promotes extracellular matrix remodelling, with increased collagen deposition and cross-linking, resulting in ventricular stiffening. These processes are further amplified by low-grade chronic inflammation, which combined with cardiometabolic comorbidities, accelerates HFpEF progression [53].

### 3.13 Diabetes and Metabolic Dysfunction

Diabetes mellitus is a major risk factor for heart failure and contributes to both HFpEF and HFrEF through metabolic, microvascular, and inflammatory mechanisms, with a particularly strong association with HFpEF [54,55]. Chronic hyperglycaemia and insulin resistance promote systemic inflammation, endothelial dysfunction, and oxidative stress, which impair nitric oxide bioavailability and disrupt myocardial relaxation.

At the myocardial level, diabetes is associated with a distinct diabetic cardiomyopathy phenotype, characterized by impaired energetics, increased myocardial stiffness, and interstitial fibrosis. These changes are driven in part by advanced glycation end-products (AGEs), which promote collagen cross-linking and extracellular matrix remodelling, as well as by lipotoxicity and mitochondrial dysfunction resulting from altered substrate utilisation [54]. Diabetes also contributes to coronary microvascular dysfunction, reducing myocardial perfusion reserve and exacerbating diastolic dysfunction. Impaired insulin signalling and metabolic inflexibility shift myocardial substrate utilization toward fatty acid oxidation, reducing energetic efficiency and increasing oxygen demand [55]. At the systemic level, diabetes interacts with other organs including the kidney, adipose tissue, and skeletal muscle to amplify HF pathophysiology. Diabetic nephropathy promotes volume overload and neurohormonal activation, while insulin resistance and adipokine dysregulation contribute to skeletal muscle dysfunction and reduced exercise capacity. Collectively, these mechanisms position diabetes as a central driver of multiorgan dysfunction and HFpEF pathogenesis.

## 4. Current Management, Guideline Recommendations and Service Delivery

### 4.1 Current Management, Guideline Recommendations

Management of heart failure across the UK and Europe is guided by the NICE and ESC respectively. Within these frameworks, echocardiography is performed to confirm the diagnosis and classify patients according to left ventricular ejection fraction as HFrEF, HFmrEF, or HFpEF [56]. This phenotypic classification is clinically important, as therapeutic strategies and evidence-based interventions differ substantially between these groups.

In patients with HFrEF, ESC guidance focuses on the use of four core drug classes, namely ARNIs (or ACE inhibitor/ARBs), beta-blockers, MRAs, and SGLT2 inhibitors. Support for SGLT2 inhibitors was strengthened in the 2023 update following evidence from trials such as DAPA-HF and EMPEROR-Reduced, which demonstrated reductions in both heart-failure admissions and cardiovascular mortality [57,58]. Beyond their demonstrated efficacy in reducing cardiovascular death and heart failure hospitalisation, SGLT2 inhibitors display practical characteristics that facilitate implementation in routine care. These agents require no dose titration, exert minimal blood pressure lowering effects, and demonstrate consistent benefit across a broad range of renal function and comorbidity burden [29]. Real-world registry data confirm favourable tolerability and adherence, including in elderly and multimorbid populations. Furthermore, cost-effectiveness analyses indicate that reductions in hospitalisation offset acquisition costs, supporting their role as foundational therapy across heart failure phenotypes [59].

Device therapy is recommended in selected patients with persistent left ventricular systolic dysfunction despite optimal medical therapy. Cardiac resynchronisation therapy (CRT), delivered as either CRT-P (cardiac resynchronisation therapy with pacing) or CRT-D (cardiac resynchronisation therapy with defibrillator), is indicated in patients with LVEF ≤35%, sinus rhythm, and a QRS duration ≥130 ms, particularly in the presence of left bundle branch block. Implantable cardioverter-defibrillators, including CRT-D devices, are recommended for the primary prevention of sudden cardiac death in patients with LVEF ≤35% and a life expectancy exceeding one year [56].

Treatment options for HFmrEF and HFpEF have historically been more limited. However, recent ESC guidance has extended the use of SGLT2 inhibitors to these groups, largely informed by the EMPEROR-Preserved trial, which demonstrated a reduction in heart-failure hospitalisations in patients with preserved ejection fraction [60]. Despite this, management in routine practice still places considerable emphasis on addressing contributory conditions such as hypertension, atrial fibrillation, diabetes, and obesity, with diuretics used primarily to relieve congestion [61].

In addition to reducing mortality and hospitalisation, contemporary disease-modifying therapies variably influence symptom burden and health-related quality of life and outcomes that are central to patients’ daily experience. A recent comparative meta-analysis evaluating major pharmacological classes demonstrated that SGLT2 inhibitors were associated with consistent and clinically meaningful improvements in patient-reported health status, including increases in KCCQ scores and reductions in symptom burden. In contrast, while ARNIs, MRAs, and beta-blockers confer substantial survival and hospitalisation benefits, their effects on exercise capacity and health-status metrics appear more modest or neutral. These findings highlight that therapeutic classes may differentially influence longevity, functional capacity, and lived patient experience, underscoring the importance of integrating validated patient-reported outcome measures into routine heart failure management [62].

In the setting of acute heart failure, NICE continues to recommend intravenous loop diuretics as first-line treatment, with supplemental oxygen reserved for patients who are hypoxic. Vasodilators may be appropriate in patients presenting with significantly raised blood pressure [6]. Importantly, contemporary evidence supports early initiation or optimisation of GDMT during acute admissions. Post-discharge vulnerability data from EVEREST and registry analyses demonstrate high event rates in the early post-hospitalisation period. The ESC additionally highlights the potential benefit of starting longer-term heart-failure therapies during the admission or shortly after discharge, where clinically appropriate [63].

### 4.2 Service Delivery

Service delivery models place increasing importance on specialist heart failure teams. HF nurses play a central role in post-discharge care, supporting medication adjustment over time and acting as a point of continuity between hospital and community services. Early follow-up after discharge is therefore a key component of NICE-recommended care pathways [61].

Despite major therapeutic advances, the implementation of evidence-based therapies into population-level benefit remains constrained by system-level challenges. Workforce limitations in cardiology and specialist heart failure nursing limit timely access to multidisciplinary care, particularly in rural and socioeconomically deprived regions. High-income healthcare systems are not immune to substantial regional variation in prescription rates of GDMT, device implantation, and access to advanced therapies, contributing to inequitable outcomes. Registries consistently demonstrate underutilisation and delayed optimisation of foundational therapies, with many eligible patients failing to receive target doses of renin-angiotensin system inhibitors, beta-blockers, mineralocorticoid receptor antagonists, or SGLT2 inhibitors. This “implementation gap” reflects structural constraints including clinic capacity, fragmented care transitions, and therapeutic inertia [64].

Digital health strategies, including telemonitoring and remote haemodynamic assessment, have been proposed as mechanisms to improve surveillance and reduce hospitalisation; however, results have been heterogeneous. While invasive pulmonary artery pressure monitoring has demonstrated reductions in heart failure hospitalisation in selected high-risk populations, non-invasive telemonitoring approaches have yielded mixed findings across trials, underscoring the importance of patient selection, system integration, and workflow design [65].

Addressing these structural barriers including workforce expansion, equitable service distribution, integration of digital tools within established care pathways, and implementation-focused quality improvement initiatives is essential to narrowing the gap between trial efficacy and real-world effectiveness. Sustainable improvement in heart failure outcomes will therefore depend not only on novel therapies but also on optimised healthcare delivery models capable of scaling evidence-based care.

Overall, contemporary heart-failure management in the UK and Europe places greater emphasis on recognising the condition early and introducing therapies sooner than in previous guideline iterations [62]. The expanding role of SGLT2 inhibitors across the ejection-fraction spectrum reflects this shift, while ongoing reliance on specialist teams highlights the importance of service organisation in translating guideline recommendations into practice.

## 5. Emerging Therapies and Precision Medicine

Recent data presented at the European Society of Cardiology Congress in 2025 highlight a rapidly evolving landscape in heart failure therapeutics, characterised by renewed interest in older agents, expanded use of established drug classes, and precision approaches targeting specific mechanisms of disease. A cardiac glycoside, digitoxin was re-examined within the DIGIT-HF trial in advanced HFrEF. Unlike digoxin, digitoxin is hepatically cleared and demonstrated reductions in all-cause death and HF hospitalisation. While detailed outcome analyses, subgroup data, and comprehensive safety evaluation remain limited, the findings suggest a potential role for this classic agent as an adjunct in patients who remain symptomatic despite full guideline-directed medical therapy [66].

In acute heart failure, the DAPA ACT HF-TIMI 68 study evaluated early in-hospital initiation of dapagliflozin. While underpowered individually and statistically neutral, its pooled analysis with EMPULSE and SOLOIST-WHF produced robust evidence that early SGLT2 inhibition reduces worsening HF and cardiovascular death, reinforcing recommendations to initiate SGLT2 inhibitors during hospitalisation rather than delaying until outpatient follow-up [67]. These data support a shift in which acute heart failure hospitalisation is reframed as a window of therapeutic opportunity. Early initiation and rapid optimisation of disease-modifying therapies may mitigate remodelling, reduce recurrent hospitalisation, and improve long-term survival, thereby altering disease trajectory rather than merely achieving short-term stabilisation.

Building on the progress of SGLT2 inhibitors, attention has increasingly turned toward therapies targeting the metabolic and inflammatory drivers of HFpEF, particularly in obesity-related phenotypes. Glucagon-like peptide-1 receptor agonists (GLP-1RAs) have recently emerged as promising therapies in HFpEF, particularly in the context of obesity and metabolic dysfunction. The landmark STEP-HFpEF trial demonstrated that once-weekly semaglutide (2.4 mg) significantly improved heart failure related symptoms, physical limitations, and exercise capacity, alongside substantial weight reduction, in patients with obesity-related HFpEF [68,69]. These findings were replicated in the STEP-HFpEF DM trial, which extended these benefits to patients with concomitant type 2 diabetes, showing improvements in quality of life, glycaemic control, and functional status [70]. Importantly, pooled analyses confirm that semaglutide consistently enhances KCCQ scores, exercise performance, and body weight, suggesting meaningful symptomatic and functional benefits across HFpEF phenotypes [69].

Mechanistically, GLP-1RAs target several central pathways implicated in HFpEF, including visceral adiposity, systemic inflammation, insulin resistance, and endothelial dysfunction, thereby addressing the cardiometabolic drivers of disease. Emerging meta-analyses further suggest that newer GLP-1RAs may reduce heart failure events and composite cardiovascular outcomes, particularly in patients with obesity or diabetes, although definitive effects on hard endpoints such as mortality and hospitaliation remain under investigation [71,72]. Collectively, these data support GLP-1RAs as a potential disease-modifying therapy in HFpEF while ongoing and future trials will determine whether these benefits translate into reductions in heart failure hospitalization and mortality, which remain key unmet needs in HFpEF.

Another demographic explored was pharmacotherapy in left ventricular assisted device (LVAD) recipients: ENVAD-HF, the first randomised study of sacubitril/valsartan in patients supported with HeartMate 3 devices, showed the drug to be safe and associated with reduced antihypertensive requirements and improved quality-of-life indices [73]. Finally, MAPLE-HCM demonstrated that the cardiac myosin inhibitor aficamten significantly improved exercise capacity, symptoms and biomarkers compared with metoprolol in obstructive hypertrophic cardiomyopathy, challenging the long-standing paradigm of β-blocker first-line therapy and signalling the rise of mechanism-based treatments in cardiomyopathies [74].

Therapies targeting the nitric oxide-cGMP pathway also featured with the VICTOR trial assessing vericiguat in stable chronic HFrEF [75]. Although its primary composite outcome was not met, cardiovascular mortality was reduced and pooled data with VICTORIA (2020) confirmed benefit in higher-risk patients [76]. Across these trials, a unifying theme emerges where HF therapy is transitioning toward earlier initiation, mechanism-specific targeting, and precision application with benefits extending to populations historically lacking evidence, such as acute HF and LVAD-supported patients. These findings underscore a future in which HF management becomes increasingly personalised, multidimensional, and biologically informed.

Large anti-cytokine strategies have had mixed results: anti-TNF agents (etanercept, infliximab; RENAISSANCE/RECOVER/RENEWAL and ATTACH programmes) failed to improve outcomes and in some arms increased risk, illustrating that broad cytokine neutralisation can be harmful or insufficiently targeted [77]. In contrast, targeted inhibition of the IL-1 pathway has yielded encouraging signals: the CANTOS trial (canakinumab, anti-IL-1β) reduced cardiovascular events in post-MI patients and, in prespecified analyses, was associated with fewer HF hospitalisations among responders [78]. Smaller HF trials of IL-1 blockade (anakinra; REDHART and follow-up studies) have shown improvements in inflammatory biomarkers and exercise capacity in selected cohorts, although results are heterogeneous and larger outcome trials are awaited [79]. Despite encouraging mechanistic rationale and improvements in inflammatory biomarkers and functional parameters in early-phase studies, heart failure-specific trials of IL-1 blockade have generally been small and heterogeneous, often relying on surrogate endpoints rather than hard clinical outcomes. Moreover, signals from larger cardiovascular outcome trials derive primarily from post-hoc or prespecified subgroup analyses, limiting definitive conclusions regarding efficacy across heart failure phenotypes.

More recent strategies target upstream inflammasome signalling (a protein complex involved in inflammation) and mitochondrial oxidative injury. The inflammasome NLRP3 inhibitor dapansutrile (OLT1177) completed early phase safety and pharmacodynamic testing in HFrEF, demonstrating tolerability and biological signals that justify larger trials [80]. Mitochondria-targeted antioxidant/peptide elamipretide (SS-31) aim to stabilise cardiolipin, reduce ROS and improve energetics. Preclinical and early-phase human studies report favourable effects on mitochondrial function and cardiac performance, however they lack generalisability and definitive outcome data remain pending [81]. Colchicine, a low-cost inhibitor of tubulin-dependent inflammasome activation reduced ischaemic events after MI (COLCOT) and is under investigation in HF populations; early HF studies show mixed signals and require phenotype-specific evaluation [82].

Early therapeutic efforts targeted the functional restoration of ATP-dependent calcium pump SERCA2a. Gene therapy using an adeno-associated virus 1 (AAV1) vector carrying the *SERCA2a* gene showed encouraging biological signals in phase 1/2 studies, but the CUPID I and subsequent CUPID II trials were neutral, largely due to limitations in vector delivery, dosing efficiency, and patient heterogeneity [83]. Importantly, these results did not invalidate calcium handling as a target but highlighted the need for improved technologies and patient stratification. Current research is revisiting *SERCA2a* gene transfer using more efficient capsids, enhanced promoters, and optimised delivery routes. The recent *Nature Medicine* phase‐1 trial of a cardiotropic AAV‐mediated gene therapy (AB-1002) for nonischaemic systolic heart failure improved these limitations with better uptake in human myocardium at a greater than three to tenfold higher dose compared with CUPID II. Over a 12-month follow-up, the therapy was well tolerated with acceptable safety and showed functional improvements and quality of life scores in most participants. These preliminary efficacy and biologic signals support continued development, including an ongoing phase 2 study (GenePHIT) [84].

Collectively, while these strategies reflect important advances in mechanistic understanding, many remain supported by early-phase or heterogeneous data, highlighting the importance of rigorous, adequately powered outcome trials before integration into routine clinical practice.

## 6. Future Directions and Conclusions

Heart failure remains one of the most complex and burdensome syndromes in contemporary cardiovascular medicine, reflecting the convergence of demographic change, multimorbidity, and improved survival from acute cardiac events. As outlined in this review, HF is not a single disease entity but a heterogeneous clinical syndrome encompassing distinct phenotypes, defined by ejection fraction, haemodynamic stress, ventricular involvement, disease chronicity, and functional limitation. These classifications are not merely descriptive as they provide a framework that links underlying pathophysiology to prognosis and therapeutic response, they remain central to guideline-directed diagnosis and management. As survival continues to improve, patient-centred outcomes including symptom burden, functional capacity, and quality of life increasingly define therapeutic success and should be prioritised alongside conventional clinical endpoints in future trials and care models.

Chronic neurohormonal activation remains the unifying paradigm in HF progression. Sustained activation of the sympathetic nervous system, renin–angiotensin–aldosterone system, and vasopressin pathways drives adverse remodelling, fibrosis, arrhythmogenesis, and multiorgan dysfunction, while the natriuretic peptide system represents an endogenous but ultimately insufficient counter-regulatory response. These insights have directly informed the development of disease-modifying therapies that now form the cornerstone of HFrEF management. The success of combined neurohormonal blockade, particularly with ARNI, beta-blockers, MRAs and SGLT2 inhibitors, represents one of the most significant advances in cardiovascular therapeutics, translating mechanistic understanding into substantial reductions in mortality and hospitalisation.

Nevertheless, major unmet needs remain. HFpEF, now the most prevalent HF phenotype in ageing populations, exemplifies the limitations of traditional models focused on systolic dysfunction. Its pathogenesis is driven less by cardiomyocyte loss and more by systemic inflammation, endothelial dysfunction, microvascular disease, and myocardial stiffening arising from cardiometabolic comorbidities. This model is also increasingly supported by recognition that HFpEF is not solely a myocardial disorder but a multiorgan syndrome, in which dysfunction of the kidney, skeletal muscle, liver, pulmonary vasculature, and adipose tissue contributes directly to haemodynamic stress, impaired cardiac reserve, and symptom burden. While SGLT2 inhibitors represent the first class to demonstrate consistent outcome benefits in HFpEF, treatment remains largely supportive, underscoring the need for phenotype-specific approaches beyond ejection fraction alone.

Emerging evidence reviewed here highlights that HF is increasingly understood as a disorder of intersecting molecular pathways, including inflammation, oxidative stress, disordered calcium handling, and genetic susceptibility. Broad anti-inflammatory strategies have largely failed, but more selective, mechanism-driven approaches such as IL-1 pathway inhibition, inflammasome modulation, and mitochondrial-targeted therapies offer renewed promise.

Disturbances in intracellular calcium cycling, long recognised as central to systolic dysfunction, are again being targeted using improved gene therapy vectors and delivery platforms, overcoming earlier translational limitations. Similarly, there is increasing recognition that therapies targeting systemic cardiometabolic dysfunction may offer a complementary approach to conventional cardiac-focused treatments. GLP-1 mimetics, originally developed for obesity and diabetes, have demonstrated improvements in symptoms, functional capacity, and quality of life in patients with HFpEF, particularly in those with obesity-related disease. These agents exert pleiotropic effects on weight, insulin resistance, inflammation, and vascular function, directly targeting key upstream primary and secondary drivers of heart failure pathophysiology. By moving beyond phenotype labels alone and focusing on the underlying pathophysiological mechanisms of therapeutic response and failure, this review provides a translational framework that complements and extends existing descriptive reviews and supports the evolution toward precision-oriented heart failure care.

Perhaps the most transformative future direction lies in the integration of genomics, biomarkers, and advanced imaging to enable precision medicine in HF. Large-scale GWAS and polygenic risk models are beginning to define inherited susceptibility across HF subtypes, while next-generation sequencing identifies monogenic cardiomyopathies with direct therapeutic and prognostic implications. Integration of polygenic risk scores and genotype-guided trial enrolment may permit biological stratification beyond phenotypic classification alone. Rather than grouping patients solely by ejection fraction, future therapeutic approaches may target dominant molecular pathways within genetically defined subgroups. Although routine genotype-guided pharmacotherapy is not yet established, precision-based strategies may enhance trial design and identify patients most likely to derive benefit from targeted interventions. Incorporation of structured genetic testing within multidisciplinary heart failure services may therefore represent an important step toward integrating molecular stratification into routine clinical care. Such an approach would permit rational selection of therapies, neurohormonal, metabolic, anti-inflammatory, or molecular, tailored to individual pathophysiological burden.
